# Body shape expectations and self-ideal body shape discrepancy in women seeking bariatric surgery: a cross-sectional study

**DOI:** 10.1186/s40608-014-0028-y

**Published:** 2014-12-24

**Authors:** Hilary I Price, Deborah M Gregory, Laurie K Twells

**Affiliations:** Clinical Epidemiology Unit, Health Sciences Centre, Faculty of Medicine, Memorial University of Newfoundland, Room 1715, 300 Prince Philip Drive, NL A1B 3V6 St. John’s, Canada; Eastern Health, Janeway Hostel, Health Sciences Centre, Patient Research Centre, 300 Prince Philip Drive, Room 533 St. John’s, NL A1B 3V6 St. John’s, Canada; School of Pharmacy, Health Sciences Centre, Memorial University of Newfoundland, Room 3445, 300 Prince Philip Drive, St. John’s, NL A1B 3V6 St. John’s, Canada

**Keywords:** Body shape expectations, Silhouette, Bariatric surgery, Laparoscopic sleeve gastrectomy

## Abstract

**Background:**

Postoperative body shape expectations (BSE) of bariatric surgery candidates remain relatively unexplored, and may have important implications for weight loss outcomes, treatment satisfaction, and education.

**Methods:**

The ‘Silhouette Figure Rating Scale’ was administered to 69 consecutive female candidates. Self-perceived current and goal body shape and postoperative BSE in four categories; “dream, “happy”, “acceptable”, and “disappointed” were examined.

**Results:**

The mean age and BMI of the sample was 43.4 ± 8.9 years and 48.8 ± 7.0 kg/m^2^. Self-ideal body shape discrepancy of 4.1 ± 1.3 silhouettes was reported, indicating body image dissatisfaction. 53% incorrectly identified the silhouette associated with their actual BMI. Goal body shape (4.3 ± 0.8 silhouettes) corresponded to a BMI figure 23.1 kg/m^2^- 26.2 kg/m^2^. The postoperative “dream” (4.1 ± 1.0 silhouettes), “happy” (5.0 ± 0.8 silhouettes), “acceptable” (5.3 ± 1.0 silhouettes), and “disappointed” (6.9 ± 1.0 silhouettes) BSE corresponded to silhouettes that were thinner than the thinnest silhouette clinically expected based on a 56.1% excess weight loss 1-year after laparoscopic sleeve gastrectomy (LSG) or a 22.3% to 47.2% total body weight loss.

**Conclusions:**

Women seeking bariatric surgery experience body image dissatisfaction and misperceive their actual body size. BSE do not correspond with evidence-based LSG weight loss outcomes.

## Background

Excess weight is a risk factor for the development of many associated comorbidities including hypertension, Type 2 diabetes, cardiovascular disease, osteoarthritis, sleep apnea, certain cancers, and premature mortality [1–3]. Bariatric (weight loss) surgery is a treatment known to promote significant, sustainable weight loss in individuals with class III obesity (BMI ≥ 40 kg/m^2^) and individuals with medically complicated class II obesity (BMI 35.0-39.9 kg/m^2^ with a major comorbidity) [4]. Laparoscopic sleeve gastrectomy (LSG) is a restrictive type bariatric surgery that promotes weight loss by reducing the stomach volume by 80%, leaving a small stomach ‘sleeve’ [2,4,5]. Average clinically expected percent excess weight loss (%EWL) 1- year after LSG is 56.1% [6]. The primary focus of bariatric surgery has been weight loss associated with these procedures; however many other psychosocial (e.g., eating disorder), health (e.g., improvement/resolution in comorbid conditions), and quality of life related benefits (e.g., mobility, self care, usual activities, pain, anxiety, depression) are observed postoperatively [4,7–18].

Body image disturbance describes an individual’s misperception of body size, inaccurate assessment of body part size, concern about body attributes, and/ or inability to determine a realistic attainable size [9,19–21]. The relationship between body image and obesity in adults and the issue of body image dissatisfaction and its clinical significance has been the focus of a number of researchers [15,22]. Studies have demonstrated that body image disturbance improves following bariatric surgery [7,16,9–14,23,24]. This has been observed despite differences in the surgical populations investigated, aspects of body image disturbance measured, survey instruments used, and study design.

Body shape expectations and self-ideal body shape discrepancy are components of body image disturbance that focus on specific expectations of body shape, and the discrepancy between perceived current body shape and ideal body shape, respectively [25,26]. Self-ideal body shape discrepancy has been used as an indicator of body image dissatisfaction [27,28].

A limited number of studies have used the Silhouette Figure Rating Scale (SFRS) to study body shape expectations and body image disturbance in adults seeking bariatric surgery [27,29]. These studies observed that body image improves after Roux-en-Y gastric bypass (RYGB) [29] and that current body image improves after bariatric surgery although patients may idealize thinner silhouettes than before surgery [27]. Rapid, surgically induced weight loss may be seen by patients as a mechanism to achieve their idealized body shape, thereby elevating their expectations of body shape change after surgery [27]. Unrealistic expectations for postoperative *weight loss* have been observed in populations seeking both non-surgical [30–38] and surgical [39–43] weight loss interventions. Studies have noted that younger, Caucasian women with higher BMIs seem to have the most unrealistic postoperative *weight loss* expectations and idealize thinner body images [37,38,41,44].

Evidence suggests that unmet expectations may negatively impact postoperative outcomes such as treatment satisfaction, weight loss, mood, and behavior maintenance [33,34,37,45,46], or motivate patients to pursue weight maintenance behaviors [30,45,47–50]. In the latter case, these negative states may contribute to weight regain after bariatric surgery, which would negate the long-term health risk reduction used to justify the surgical risk and expense associated with this procedure. An understanding of the goals and expectations of surgical candidates is therefore of critical importance to patient care and treatment outcomes, and should be included in the discussion of surgical risks and benefits.

To the best of our knowledge, no research findings have been published that profile the *body shape expectations* of bariatric surgery candidates using the SFRS.

Therefore the aim of the current study was to describe the body shape expectations and self-ideal body shape discrepancy using the SFRS in women seeking LSG surgery in Newfoundland and Labrador (NL), Canada.

## Methods

### Participants and setting

Female candidates for bariatric surgery in attendance at a mandatory LSG education session in NL between October 2011 and March 2012 (n = 69) were eligible for inclusion in this cross-sectional survey study. There was an insufficient sample size to support meaningful conclusions about males in this sample population (n = 7). Individuals were invited to attend the education session if the bariatric nurse practitioner determined them to be eligible for bariatric surgery based on screening of their physician referral form and the Canadian clinical practice guidelines [4]. All eligible candidates agreed to complete the questionnaire before the start of the education session. All candidates agreed to participate in this research study. Ethical approval was obtained from the Human Research Ethics Authority of Newfoundland and Labrador before data collection commenced.

### Survey instruments

This study evaluated different levels of body shape expectation and self-ideal body shape discrepancy using a combination of two reliable and valid instruments as a review of the literature did not yield any existing tool that could measure these constructs. Therefore, the ‘Goals and Relative Weights Questionnaire’ (GRWQ) [34] and the validated ‘Silhouette Figure Rating Scale’ (SFRS) [51] were used to achieve this aim.

The GRWQ was developed and validated [34] to further the understanding of patient’s goals, expectations, and evaluations of behavioral weight loss therapy. Part II of the questionnaire asks participants to numerically define their postoperative *weight loss expectations* in the categories “dream”, “happy”, “acceptable”, and “disappointed” weight. Simple alteration of the GRWQ definitions was necessary to put them into context for bariatric surgery candidates. These modifications were made to suit the study population according to similar studies in the clinical literature [42,43]. The modified weight loss expectation categories and descriptions are presented in Table 1.

**Table 1:** **Weight loss expectation category descriptions** [34]

**Weight loss expectation**	**Description**
**Dream**	A weight that you would choose if you could weigh whatever you wanted after weight loss surgery.
**Happy**	This weight is not as ideal as your dream weight. It is a weight, however, that you would be happy to achieve from weight loss surgery.
**Acceptable**	A weight that you would not be particularly happy with, but one that you would accept after weight loss surgery, since it is less than your current weight.
**Disappointed**	A weight that is less than your current weight, but one that you could not view as successful in any way. You would be disappointed if this was your final weight after weight loss surgery.

The SFRS is a series of nine gendered silhouettes of progressively larger body size used to quantitatively assess *body shape expectation* and the degree and direction of self-ideal body shape discrepancy [51]. It has been psychometrically validated with two-week test-retest reliability (r = 0.55-0.92, p < 0.001) and small to moderate correlations with other measure of body image disturbance, eating disturbance, and overall self-esteem (r = 0.16-0.60, p < 0.01) [52]. The SFRS has been used to evaluate body image in populations of individuals with overweight and obesity [12,27,53,54] despite some methodological concerns relating to the use of silhouettes to measure body image [55].

Participants were asked to indicate their “dream”, “happy”, “acceptable”, and “disappointed” postoperative body shape expectations by indicating the figure that they associated with the GRWQ expectation definition in each category. Perceived current body shape and goal body shape were also determined. Goal body shape expectations were assessed with the survey prompt “Circle your goal body figure” and were not related to any recommendation or counseling provided, this was a personal body shape goal.

All data were self-reported. This included all demographic (date of birth, sex, marital status, highest level of education, and employment status), height, and weight related data. Comorbidities were self-reported in response to the question “Have you ever been diagnosed by a doctor with any of the following medical conditions?”.

### Statistical analyses

Data were analyzed using SPSS 19.0 (SPSS IBM, New York, USA). Demographic variables and body shape expectations were analyzed using descriptive statistics and a p-value of 0.05 was considered statistically significant. The %EWL was calculated according to the equation:%EWL = (current weight–weight loss expectation category weight) ÷ (current weight– ideal body weight) × 100 [56]. The SFRS was evaluated on a 1–9 scale (1 = leanest silhouette to 9 = largest silhouette).

Self-ideal body shape discrepancy was calculated as the difference between participants’ mean current body shape and mean goal/ideal body shape using the equation: self-ideal body shape discrepancy = current shape- ideal shape [25]. In this study we used the terminology goal body shape interchangeably with ideal body shape. This was done to facilitate integrations of the SFRS and the GRWQ scales. “Goal” was assumed to be equivalent to “ideal” body shape based on comparison of findings of “ideal” body shape in the literature [27]. In the context of this study, a “goal” body shape was a personal goal chosen by each woman before attending pre-surgical education or counseling of any kind for bariatric surgery.

### Comparison of SFRS silhouette and BMI data

A number of researchers have modified the expectation definition wording to suit a bariatric surgery seeking population [41–43]. The SFRS was modified with permission for use in this study. Previous studies have established BMI values associated with each female SFRS silhouette [57–59], which were superimposed above each body figure in the current study. The obesity classes associated with each figure’s assigned BMI value was determined according to the Canadian clinical practice guidelines [4] and was also displayed above the figure. Thus, a modified visual scale with the ability to display both SFRS silhouette data (1 = leanest to 9 = largest silhouette) and BMI data (kg/m^2^) was created.

**Figure 1:**
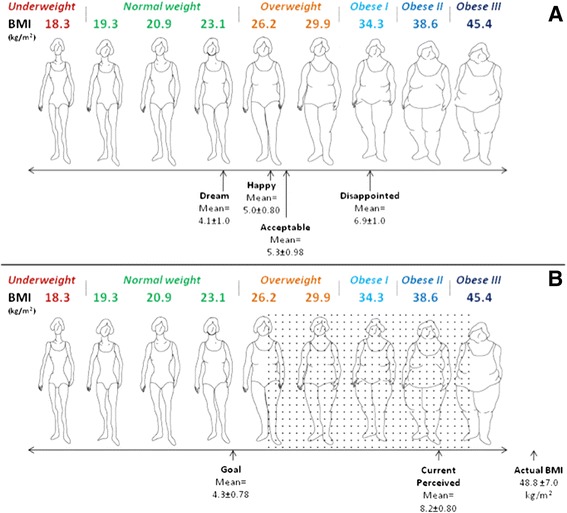
**Body shape expectations of women seeking laparoscopic sleeve gastrectomy compared to average clinically expected %EWL 1-year post surgery. A**. depicts the silhouettes associated with women’s “dream”, “happy”, “acceptable”, and “disappointed” postoperative body shape expectations. **B**. depicts the goal, current perceived, and actual self-reported silhouettes of women seeking LSG compared to the range of clinically expected body shapes 1-year post bariatric surgery. Dotted area indicates the range of silhouettes clinically expected 1-year after LSG based on a 56.1%EWL in this sample of women [6]. BMI values assigned according to population normative data [57]. Obesity classes arranged according to Canadian Clinical Practice Guidelines [4]. n = 69, less than 10% missing data. Reprinted with permission from Stunkard AJ, Sorenson T, Schulsinger F: Use of the Danish Adoption Register for the study of obesity and thinness. In *The Genetics of Neurological and Psychiatric Disorders*. Edited by Kety SS, Rowland LP, Sidman RL, Matthysse SW. New York: Raven Press; 1983: 115–120.

In Figure 1A, candidates’ “dream”, “happy”, “acceptable” and “disappointed” body shape expectations were displayed on the modified scale to allow for visual estimation of the BMI and obesity class associated with each of these expectation categories. In Figure 1B, the women’s mean actual BMI, calculated based on self-reported height and weight data, was displayed with the mean current perceived and goal body shapes to provide a visual reference to candidates’ current weight status compared to their body perceptions. The range of postoperative BMIs that would result if the leanest woman and the largest woman in the current study sample each achieved a 56.1%EWL was calculated and also displayed in this figure. The range of postoperative BMIs estimated based on these calculations was superimposed onto the modified scale using the silhouettes’ assigned BMI values, and was indicated by a stippled region overlaying the scale. Superimposing the range of clinically expected weight loss 1-year after LSG for this sample of women over the female SFRS silhouettes in this way was performed to facilitate a visual comparison of candidates’ body shape expectations and the body shapes that the clinical evidence supports that they might achieve 1-year post LSG.

## Results

Women seeking bariatric surgery in the current NL sample had an average age of 43.4 ± 8.9 years and weighed 131.0 ± 19.7 kg (288.8 ± 43.4 lbs) (Table 2). The average BMI was 48.8 ± 7.0 kg/m^2^. The majority of participants were employed full-time; although a high proportion (19.1%) were on short or long term disability leave. Most women (59.4%) had completed post-secondary education.

**Table 2:** **Demographic characteristics of women seeking bariatric surgery**

**Characteristic**	**Mean ± SD**	
Age, years	43.4 ± 8.9	
Weight, kg	131.0 ± 19.7	
BMI, kg/m^2^	48.8 ± 7.0	
Number of chronic conditions	2.8 ± 2.1 (median 2.0)	
	n	%
Married	47	68.1
Employment status		
Employed full-time	31	45.6
Disability leave (short & long term)	13	19.1
Unemployed	7	10.3
Other employment (part-time, casual, home-maker, retired, other)	17	25.0
Education status		
Completed post-secondary	41	59.4
Some post-secondary	11	15.9
High school diploma or less	17	24.7

^a^n = 69, all data self-reported.

Study participants had a self-ideal body shape discrepancy of 4.1 ± 1.3 silhouettes, indicating considerable discrepancy between their self-perceived current body image and the image of their ideal body. The positive nature of this value indicates that women idealized a thinner silhouette along the figure rating scale.

The actual BMI of women seeking LSG was 48.8 ± 7.0 kg/m^2^. The largest female silhouette on the SFRS is associated with a BMI of 45.4 kg/m^2^, indicating that woman in this sample had a silhouette larger than the largest silhouette depicted on the SFRS. The majority (53%) of candidates incorrectly identified the silhouette associated with their actual BMI (self-perceived current body shape = 8.2 ± 0.8 silhouettes) by under-estimating their true size.

Study participants reported “dream”, “happy”, “acceptable”, and “disappointed” postoperative body shapes of 4.1 ± 1.0 silhouettes, 5.0 ± 0.8 silhouettes, 5.3 ± 1.0 silhouettes, and 6.9 ± 1.0 silhouettes, respectively (Figure 1A). Participants set their body shape goal after bariatric surgery at 4.3 ± 0.8 silhouettes, and perceived current body shape to correspond to silhouette 8.2 ± 0.8 (Figure 1B). This corresponded to a desired weight loss of between 30.0 ± 17.2 kg for a “disappointed” weight and 62.7 ± 18.0 kg for a “dream” weight, or a 22.3 ± 11.9% to 47.2 ± 8.5% loss of total body weight from bariatric surgery.

The relationships between current body shape, goal body shape, and evidence-based 1-year weight loss outcomes from LSG were explored (Figure 1B). In this sample, achievement of a clinically expected 56.1%EWL 1-year after LSG would correspond to postoperative BMIs between 26.8 kg/m^2^- 39.9 kg/m^2^. It was observed that women’s postoperative goal body shape corresponded to a silhouette with a BMI between 23.1 kg/m^2^- 26.2 kg/m^2^, suggesting it is different than the range of BMI’s corresponding with evidence-based 1-year weight loss outcomes from LSG.

## Discussion

In the current study, women seeking bariatric surgery experienced preoperative body image dissatisfaction. The majority of participants underestimated their current body size. Women’s postoperative body shape expectations did not correspond with clinically expected 1-year weight loss outcomes following LSG surgery.

To the best of our knowledge, no research findings have been published profiling the postoperative body shape expectations of women seeking bariatric surgery using the Silhouette Figure Rating Scale. The limited research available focused exclusively on the self-perceived current body shape and goal/ideal postoperative body shape expectations and did not include the “dream”, “happy”, “acceptable”, and “disappointed” body shape expectation categories [27,29].

Similar to other researchers we found that women seeking bariatric surgery experienced preoperative body image dissatisfaction [8,27,29]. High self-ideal body shape discrepancy (i.e. the difference between current self-perceived and ideal body shapes) has been associated with increased dissatisfaction with body image [25]. In their prospective survey analysis of changes in desired body shape after bariatric surgery, Munoz and colleagues [27] reported that RYGB patients had a baseline self-ideal body shape discrepancy of 4.16 ± 1.75 silhouettes. The authors concluded that this discrepancy indicated poor satisfaction with preoperative body image, which improved 1-year postoperatively. The self-ideal body shape discrepancy observed in the present study (4.1 ± 1.3 silhouettes) was similar to those reported by Munoz et al. [27], indicating that women in this study were also dissatisfied with their preoperative body image. The psychosocial pressures of prejudice, bias, and stigmatization associated with excess weight have been shown to negatively impact the mental and emotional health of obese individuals [7,60]. Bariatric care teams may also want to consider the impact of profound dissatisfaction with body image on the preoperative health of their patients.

The current study’s findings suggest that LSG candidates in NL have impaired body image satisfaction before surgery. Other studies in bariatric surgery populations also provide evidence to support this conclusion [15,16]. Neven and associates [29] reported low body image satisfaction before RYGB surgery, which improved after bariatric surgery intervention.

Using the SFRS, over half of the current study participants (53%) underestimated their current body size. The average calculated BMI, based on self-reported height and weight, was 48.8 ± 7.0 kg/m^2^ and corresponded to a silhouette larger than the largest silhouette on the scale. It has been suggested that by underestimating their current body size, these women may not be aware of the increased health risk associated with their current BMI classification [55,61].

Finally, participants’ postoperative body shape expectations did not correspond with evidence-based 1-year weight loss outcomes from LSG surgery. Participants in the current study could at best “accept” or be “disappointed” with their clinically expected postoperative body shape. In the only identified study of body shape expectations using the SFRS, Munoz and colleagues [27] observed that the ideal body silhouette of RYGB patients was 4.13 ± 0.74 (1 = leanest silhouette, 9 = largest silhouette), a value which is comparable to the postoperative “dream” (4.1 ± 1.0 silhouettes) and goal (4.3 ± 0.78 silhouettes) body shape expectations observed in the present study. Munoz et al. [27] concluded that RYGB patients have unrealistic postoperative body shape expectations. Consistent with our study findings, Neven et al. [29] also concluded that bariatric surgery patients have unrealistic body shape expectations. Unrealistic postoperative body shape expectations may negatively impact postoperative outcomes such as treatment satisfaction, weight loss, mood, and behavior maintenance [33,34,37,45,46]. Alternatively, patients’ body shape expectations may motivate them to pursue weight maintenance behaviors [30,45,47–50]. If surgery is perceived to be unsuccessful from the patient’s perspective, this may have potential psychological and emotional clinical implications that could possibly be addressed with further education and counseling.

Study strengths included the consecutive recruitment of study participants with a 100% response rate thereby eliminating the possibility of selection bias and ensuring a representative sample of LSG candidates was captured. Limitations associated with this study are the small sample size and the cross-sectional study design. This study was limited by several factors inherent in a cross-sectional study design. Self-report bias may have influenced response to survey items. Individuals tend to overestimate their height and underestimate their weight when self-reporting these data [62]. Therefore, weight estimates in this study may have been conservative, indicating that body shape expectations may be even more divergent from clinical expectations than originally thought. Referral to the Bariatric Surgery Clinic by primary healthcare physicians and specialists may have been influenced by referral bias, potentially limiting the generalizability of the study population. This study did not control for other factors that could impact body image or shape dissatisfaction, such as history of physical trauma or musculoskeletal disorders. Finally, to the best of our knowledge, no other research study has been published using this particular method or describing the postoperative body shape expectations of bariatric surgery candidates. It was therefore difficult to compare and contrast our study findings with the literature.

As the prevalence of morbid obesity [63] and the demand for bariatric surgery continue to rise [5,64,65] an understanding of the postoperative body shape expectations of bariatric surgery patients will become increasingly important. Future research should focus on replicating these findings in other populations and determining participants’ attitudes about their preoperative body shape expectations 1-year after surgery. Prospective cohort studies are needed to elucidate the relationship between preoperative expectations of body shape and postoperative outcomes.

## Conclusions

Women seeking bariatric surgery experienced preoperative body image dissatisfaction and underestimated their actual body size. Postoperative body shape expectations did not correspond with clinically expected 1-year weight loss outcomes following LSG surgery.
